# Microarray analysis in B cells among siblings with/without MS - role for transcription factor TCF2

**DOI:** 10.1186/1755-8794-1-2

**Published:** 2008-01-31

**Authors:** Jagannadha R Avasarala, Sridar V Chittur, Ajish D George, John A Tine

**Affiliations:** 1 Multiple Sclerosis Specialty Care, Kansas Neurological Consultants, PA, Wichita, KS 67218, USA; 2 Center for Functional Genomics and Department of Biomedical Sciences, University at Albany, SUNY, Rensselaer, NY 12144, USA

## Abstract

**Background:**

We investigated if global gene expression and transcription networks in B-lymphocytes of siblings with multiple sclerosis (MS) were different from healthy siblings.

**Results:**

Using virus-transformed immortalized B cells and human whole genome bioarrays with validation using RT-qPCR, we found that in siblings with MS, genes for CXCL10, serpin B1 and FUT4 were up regulated whereas CDC5L, TNFRSF19 and HLA-DR were down regulated, among others; transcription analysis showed two intersecting clusters of transcriptional factors - the larger, governed by the upregulated transcription factor 2 (TCF2) and the smaller network regulated by the downregulated CDC5L.

**Conclusion:**

No study has linked TCF2 to MS and to better understand the role of TCF2 in MS, studies in larger cohorts are required.

## Background

MS is a complex genetic disease associated with inflammation predominantly in the white matter of brain and spinal cord. It is thought to be mediated by autoreactive T cells [1,2]. Susceptibility to MS is determined by both inherited and non-inherited factors [3]. Approximately 15-20% of MS patients have a family history of MS, but large extended pedigrees are uncommon. Studies in twins [4,5] and conjugal pairs [6] show that much of the familial clustering is the result of shared genetic risk factors.

MS susceptibility is linked to HLA-DR2 [7]. Increased risk of MS in women has been detected with interleukin-1 receptor antagonist (IL-1RA) allele 2 [8], 5G5G genotype of plasminogen activator inhibitor 1 (PAI-1) gene [9] and interaction between estrogen receptor 1 (ESR1) and HLA-DR2 [10]. B cells are implicated in MS and have been found in the cerebrospinal fluid (CSF) of MS patients [11]. Additionally, oligoclonal bands identified in CSF point to the role of B cells in MS pathogenesis [12]. Furthermore, antibody-secreting B cells contribute to tissue injury [13]. We hypothesized that B cells derived from MS patients could harbor genes that confer a higher MS risk as compared to B cell gene expression in healthy siblings.

Human B-cells have a receptor for Epstein-Barr virus (EBV) and can become immortalized after in vitro infection with EBV. Additionally, the link between EBV and MS is highly impressive [14] though inconclusive; we hypothesized that analysis of gene expression and transcription networks in EBV-transformed B cells between siblings with and without MS could yield important clues to understanding the pathology of MS.

Large-scale analyses of transcripts from peripheral blood cells or brain lesions from MS patients have created possibilities for therapeutics [15] and global gene expression analysis using microarrays is a sensitive method to investigate molecular heterogeneity [16]. In this study, we tested a new software tool that is in development, to map transcription networks in the microarray data.

Our objectives were to i) to determine if gene expression and transcription networks in B-lymphocytes of siblings with MS were different from healthy siblings in EBV-transformed B cells and ii) to validate data using qPCR techniques.

## Methods

EBV-transformed B cell lines from Coriell Institute for medical research (Camden, NJ, USA) and the National Institute of General Medical Sciences (NIGMS, Bethesda, MD) were obtained for our study. As shown in Table 1, B cells were harvested from one family (# 2108, proband, affected sister) and an unaffected brother (control); cells from another family (# 2112, proband, affected brother) and three unaffected brothers (controls). Cells from the third family (# 2102) comprised of proband and an affected sister but no unaffected controls. None of the patients were on immunomodulatory agents (IMAs) to treat MS.

**Table 1:** Cell lines obtained from Corielle for our study.

**Sample ID**	**Relation**	**Family**	**Affected**	**Sex**	**Age**
GM8923	Proband	2108	Yes	Male	38
GM8922A	Sister	2108	Yes	Female	43
GM8830	Proband	2102	Yes	Female	47
GM8839	Sister	2102	Yes	Female	39
GM9013	Proband	2112	Yes	Male	35
GM9016	Brother	2112	Yes	Male	38
GM9018	Brother	2112	No	Male	42
GM9017	Brother	2112	No	Male	45
GM9023	Brother	2112	No	Male	47
GM8921A	Brother	2108	No	Male	46

Cells were harvested in 4 ml of Tri-Reagent (Molecular research center, Inc) and the total RNA was isolated using standard protocols. Briefly, B-cells were incubated at 37°C and 5% CO_2 _overnight, the day after cells were counted. Cells were expanded in RPMI 1640 with 2 mM L-glutamine and 15% fetal bovine serum following Coriell's lymphoblast line maintenance protocols. 2 vials were frozen and 1 × 10^7 ^cells were spun down and resuspended in 2 ml of Tri-reagent. The cell mixture was frozen at -80°C until further analysis. The RNA was isolated using the standard Tri-reagent protocol and further cleaned up using RNeasy kits (Qiagen) using a protocol that included an on column Dnase step. RNA samples were checked for quality on a Bioanalyzer and a nanodrop spectrophotometer. Samples with 28s/18s ratio > 1.8 were deemed acceptable for the microarray experiment. The total RNA (1 ug) was first converted to double stranded cDNA using the standard Codelink protocol. This was then purified on Qiaquick PCR columns and in vitro transcribed to labeled cRNA using biotin-11-UTP (Perkin Elmer). The labeled cRNA was purified on Qiagen RNeasy Mini columns and 10 ug was fragmented as per the Codelink protocol following which it was hybridized to the Codelink Human Whole Genome bioarrays for 18 h at 37°C. The arrays were subsequently washed, stained with Cy5-Streptavidin (GE Amersham) and scanned on an Axon Genepix 4000B scanner. The data were collected using Codelink Expression Analysis software and analyzed using Genespring software version 7.3.1 (Agilent). All microarray data are deposited at Gene Expression Omnibus as GSE 10064 and linked via [17].

### Microarray data analysis

Values below 0.01 were set to 0.01. Each measurement was divided by the 50th percentile of all measurements in that sample. All samples were normalized to the median of the control samples. Each measurement for each gene in those specific samples was divided by the median of that gene's measurements in the corresponding control samples. The gene list (54,902 transcripts) was filtered using the cross gene error model for replicates to select genes with good signal-to-noise ratios (31, 168 genes). This gene list was subjected to a student's t-test (p-value < 0.05) and yielded 1, 417 genes with significant differences between the two conditions. This list was further filtered for confidence using a Benjamini-Hochberg false discovery rate correction to obtain 1405 genes. A 1.5-fold cut-off filter was then applied to identify genes that were preferentially upregulated (260) or downregulated (452) genes in samples with disease as compared to controls. These 712 genes were further filtered to only select those which were consistently 'absent" or "present" in 3 of 4 controls and 5 of 6 diseased samples. The final gene list had 705 genes; 259 upregulated and 446 downregulated genes while using 2-fold cut off resulted in 84 upregulated and 111 downregulated genes (Tables 2 &3).

**Table 2:** Representative upregulated genes (out of 259 total).

**Probe ID**	**Fold Change**	**Common**	**Genbank**	**Description**
**Upregulated genes**				
GE53598	5.88	TINAG	NM_014464	tubulointerstitial nephritis antigen, complete cds
GE59642	5.77	CXCL10	NM_001565	gamma-interferon inducible early response gene (with homology to platelet proteins)
GE57550	4.74	CRYM	NM_001888	mu-crystallin mRNA, complete cds
GE518123	4.27	IGLL1	NM_020070	immunoglobulin lambda-like polypeptide 1 (IGLL1), transcript variant 1, mRNA
GE58736	4.20	DACT1	NM_016651	dapper homolog 1, antagonist of beta-catenin (xenopus) (DACT1), mRNA
GE79033	4.15	CD9	NM_001769	CD9 antigen (p24) (CD9), mRNA
GE55346	3.57	LRRC16	NM_017640	leucine rich repeat containing 16 (LRRC16), mRNA
GE61239	3.50	SERPINB1	NM_030666	serine (or cysteine) proteinase inhibitor, clade B (ovalbumin), member 1 (SERPINB1), mRNA
GE62781	3.50	MT1F	NM_005949	metallothionein 1F (functional) (MT1F), mRNA
GE87501	3.49	FXYD2	NM_001680	FXYD domain containing ion transport regulator 2 (FXYD2), transcript variant a, mRNA
GE61946	3.26	ARHGAP6	NM_001174	Rho GTPase activating protein 6 (ARHGAP6), transcript variant 2, mRNA
GE56932	3.22	EGLN3	NM_022073	egl nine homolog 3 (C. elegans) (EGLN3), mRNA
GE61093	3.18	ACTN1	NM_001102	actinin, alpha 1 (ACTN1), mRNA
GE87626	2.97	OSR2	NM_053001	odd-skipped-related 2A protein (OSR2), mRNA
GE59655	2.93	ATP1B1	NM_001677	ATPase, Na+/K+ transporting, beta 1 polypeptide (ATP1B1), mRNA
GE58575	2.91	PTPLA	NM_014241	protein tyrosine phosphatase-like (proline instead of catalytic arginine), member a (PTPLA), mRNA
GE57767	2.84	COL9A3	NM_001853	collagen, type IX, alpha 3 (COL9A3), mRNA
GE57621	2.83	LGALS3BP	NM_005567	lectin, galactoside-binding, soluble, 3 binding protein (LGALS3BP), mRNA
GE81389	2.82	CCR6	NM_004367	chemokine (C-C motif) receptor 6 (CCR6), transcript variant 1, mRNA
GE79898	2.81	LYPLA3	NM_012320	lysophospholipase 3 (lysosomal phospholipase A2) (LYPLA3), mRNA
GE62292	2.79	CPXM	NM_019609	carboxypeptidase X (M14 family) (CPXM), mRNA
GE57547	2.66	TCN2	NM_000355	transcobalamin II; macrocytic anemia (TCN2), mRNA
GE81525	2.52	IFI27	NM_005532	ISG12 protein
GE79641	2.48	FUT4	NM_002033	fucosyltransferase 4 (alpha (1,3) fucosyltransferase, myeloid-specific) (FUT4), mRNA
GE81272	2.47	VIM	NM_003380	vimentin (VIM), mRNA
GE57737	2.45	ENPP2	NM_006209	autotaxin mRNA, complete cds
GE80150	2.38	ELF3	NM_004433	E74-like factor 3 (ets domain transcription factor, epithelial-specific) (ELF3), mRNA
GE58207	2.34	ENG	NM_000118	endoglin (Osler-Rendu-Weber syndrome 1) (ENG), mRNA
GE57048	2.33	CCR1	NM_001295	chemokine (C-C motif) receptor 1 (CCR1), mRNA
GE57883	2.28	SELL	NM_000655	selectin L (lymphocyte adhesion molecule 1) (SELL), mRNA
GE60214	2.27	NRGN	NM_006176	neurogranin (protein kinase C substrate, RC3) (NRGN), mRNA
GE498904	2.22	DACT1	NM_016651	dapper homolog 1, antagonist of beta-catenin (xenopus) (DACT1), mRNA
GE81340	2.17	PSTPIP1	NM_003978	proline-serine-threonine phosphatase interacting protein 1 (PSTPIP1), mRNA
GE58931	2.16	TCF8	U12170	zinc finger homeodomain protein mRNA, complete cds
GE61464	2.15	SERPINB9	NM_004155	serine (or cysteine) proteinase inhibitor, clade B (ovalbumin), member 9 (SERPINB9), mRNA
GE82947	2.12	SNX22	NM_024798	sorting nexin 22 (SNX22), mRNA
GE60134	2.09	UPP1	NM_003364	uridine phosphorylase (UP), transcript variant 1, mRNA
GE54152	2.07	APOL1	NM_003661	apolipoprotein L, 1 (APOL1), transcript variant 1, mRNA
GE56786	2.02	DUSP4	NM_001394	dual specificity phosphatase 4 (DUSP4), transcript variant 1, mRNA
GE54528	2.02	TNFSF12;	NM_003809	tumor necrosis factor (ligand) superfamily, member 12 (TNFSF12), transcript variant 1, mRNA
GE61789	2.02	TDE2L	NM_178865	tumor differentially expressed protein 2 (TDE2), mRNA
GE61399	2.01	LLT1	NM_013269	lectin-like NK cell receptor (LLT1), mRNA

Fold change = diseased/normal

**Table 3:** Representative downregulated genes (out of 446 total)

Probe ID	Fold change	Common	Genbank	Description
Down regulated genes				
GE54030	0.09	EIF1AY	NM_004681	eukaryotic translation initiation factor 1A, Y-linked (EIF1AY), mRNA
GE79500	0.1	RPS4Y	NM_001008	ribosomal protein S4, Y-linked (RPS4Y), mRNA
GE54029	0.12	DDX3Y	NM_004660	DEAD/H (Asp-Glu-Ala-Asp/His) box polypeptide, Y chromosome (DBY), mRNA
GE59227	0.12	JARID1D	NM_004653	Smcy homolog, Y-linked (mouse) (SMCY), mRNA
GE55836	0.15	MLAT4	NM_018192	myxoid liposarcoma associated protein 4 (MLAT4), mRNA
GE54974	0.19	TNFRSF19	NM_148957	tumor necrosis factor receptor superfamily, member 19 (TNFRSF19), transcript variant 2, mRNA
GE788416	0.28	KRTAP4-9	AJ406941	partial mRNA for keratin associated protein 49 (KRTAP4.9 gene)
GE489986	0.32	ZFY	NM_003411	zinc finger protein, Y-linked (ZFY), mRNA
GE58359	0.35	GCHFR	NM_005258	GTP cyclohydrolase I feedback regulatory protein (GCHFR), mRNA
GE87376	0.36	BAIAP2L1	NM_018842	insulin receptor tyrosine kinase substrate (LOC55971), mRNA
GE561268	0.39	FOXD4L2	NM_199135	FOXD4-like 2 (FOXD4L2), mRNA
GE54486	0.4	DAPK2	NM_014326	death-associated protein kinase 2 (DAPK2), mRNA
GE79999	0.4	CAMK1	NM_003656	calcium/calmodulin-dependent protein kinase I (CAMK1), mRNA
GE58178	0.4	KAL1	NM_000216	Kallmann syndrome (KAL) mRNA, complete cds
GE82446	0.4	BAIAP2L1	NM_018842	insulin receptor tyrosine kinase substrate (LOC55971), mRNA
GE58327	0.41	CD83	NM_004233	CD83 antigen (activated B lymphocytes, immunoglobulin superfamily) (CD83), mRNA
GE567535	0.42	NGFRAP1	AF187064	p75NTR-associated cell death executor (NADE) mRNA
GE62339	0.42	CSE-C	NM_018978	sialic acid-specific 9-O-acetylesterase I mRNA, complete cds
GE82815	0.42	FA2H	NM_024306	fatty acid 2-hydroxylase (FA2H), mRNA
GE82367	0.44	LAPTM4B	NM_018407	lysosomal associated protein transmembrane 4 beta (LAPTM4B), mRNA phosphodiesterase 4A, cAMP-specific (phosphodiesterase E2 dunce homolog, Drosophila)
GE81609	0.45	PDE4A	NM_006202	(PDE4A), mRNA
GE61123	0.45	ZNF239	NM_005674	zinc finger protein 239 (ZNF239), mRNA
GE53189	0.46	AUTS2	NM_015570	autism susceptibility candidate 2 (AUTS2), mRNA
GE81426	0.46	USP9Y	NM_004654	ubiquitin specific protease 9, Y chromosome (fat facets-like Drosophila) (USP9Y), mRNA
GE63274	0.48	CENTG3	AL537305	FETAL BRAIN cDNA clone CS0DF025YC08 5-PRIME
GE83170	0.48	BCL2L14	NM_030766	apoptosis regulator BCL-G (BCLG), transcript variant 2, mRNA
GE58554	0.49	PRO1914	NM_014106	PRO1914 protein (PRO1914), mRNA
GE86889	0.49	SLC26A7	NM_052832	solute carrier family 26, member 7 (SLC26A7), transcript variant 1, mRNA
GE81520	0.49	BCAT1	NM_005504	branched chain aminotransferase 1, cytosolic (BCAT1), mRNA

Fold change = diseased/normal

### Hierarchical clustering

**Figure 1:**
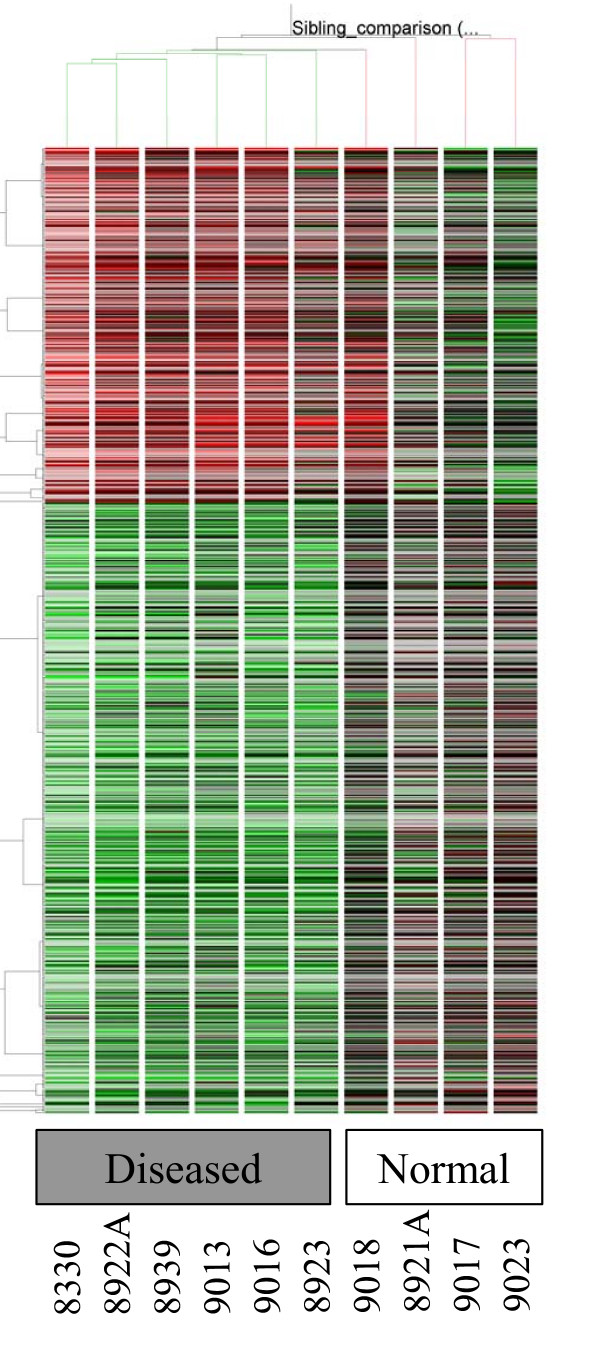
**Hierarchical clustering of all statistically significant genes that show differential expression between diseased and normal samples**. Upregulated are colored red and downregulated genes are colored green. Clustering of genes is shown on the left while condition based tree is shown on the top.

This gene list was subjected to a hierarchical clustering routine in GeneSpring using a Pearson correlation similarity measure across conditions and a standard correlation similarity measure across genes. The final tree is represented in Figure 1.

### Pathway Analysis

The 1.5 fold list was further analyzed using Onto-Express and Pathway-Express, part of the Onto-Tools package available from the Draghici laboratory. This software allows for identification of pathways that show enrichment in the microarray data and is a novel tool that translates lists of differentially regulated genes into functional profiles characterizing the impact of the condition studied.

### Quantitative PCR validation

To verify expression data obtained by the Codelink analysis, Taqman gene expression assays were obtained (Applied Biosystems) to evaluate the expression of selected genes by RT-qPCR. The assays utilized were as follows: TCF2 (Applied Biosystems assay Hs01001609_m1), CXCL10 (Hs00171042_m1), FUT4 (Hs00275643_s1), SERPINB1 (Hs00229084_m1), CD9 (Hs01124026_m1), LYPLA3 (Hs00202399_m1), SERPINB9 (Hs00394497_m1), CD83 (Hs00188486_m1), CDC5L (Hs00904622_m1), TNFRSF19 (Hs00218634_m1), HLA-DRA (Hs00219578_m1). All assays are designed to detect the appropriate transcripts at an exon-exon junction to ensure the detection of mRNA and not genomic DNA.

The expression levels of these 11 genes were evaluated by two-step Taqman-based RT-qPCR. First strand cDNA was synthesized with M-MLV reverse transcriptase (Ambion, Inc., Austin, TX) and primed with oligo-dT per the manufacturer's specifications. The cDNA equivalent of 20 ng starting RNA was then included in reactions containing 1× Taqman Master Mix (Applied Biosystems, Foster City, CA), and 1× gene-specific assay reagents as recommended by the manufacturer (Applied Biosystems). All reactions were run in triplicate. Reactions that did not contain template cDNA were included as negative controls.

Reaction plates were processed on an Applied Biosystems 7900 HT Sequence Detection System. The AmpliTaq Gold polymerase was activated at 95°C for 10 min followed by 40 cycles consisting of denaturation for 15 seconds at 95°C and annealing and extension for 60 seconds at 60°C.

Amplification data was analyzed with the ABI Prism SDS 2.1 software (Applied Biosystems). Although run individually, samples GM8921A, 9017, 9018, and 9023 were pooled for analysis as the control sample, and samples GM8830, 8922A, 8923, 8839, 9013, and 9016 were pooled for analysis as the MS sample, unless otherwise noted. Relative quantification of gene expression was performed by the ΔΔCt method [18]. GAPDH expression was used as an endogenous control to normalize expression within each sample.

### Transcription network analysis

We employed a software program that one of our authors continues to develop (G.A.) to recast the matrix of regulatory interactions found in a prior step as a directed graph overlaying color information for gene responses. Our regulatory network was constructed by integrating transcription factor binding site information (from TRANSFAC) with gene expression data using graph TFs (Bioconductor functions for transcription network analysis). The standard microarray experiments cannot measure the transcription factor activities (TFAs) directly, since TFAs are subject to post-translational modifications or ligand binding in order to exert their function.

**Figure 2:**
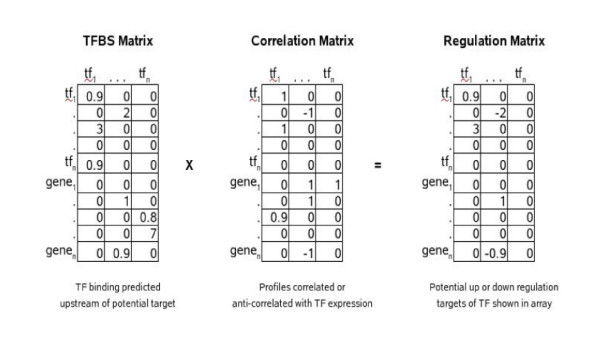
Analysis of transcription networks from microarray data.

**Figure 3:**
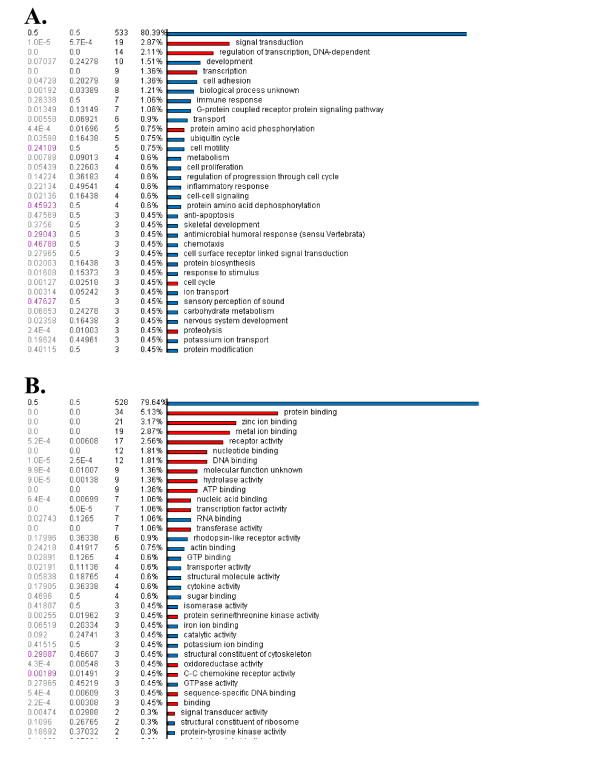
**Transcription factor network deciphered from genes differentially expressed at a 1.5 fold cutoff in microarray data**. The color 'red" shows upregulation while "green" shows downregulated expression. Genes are depicted by circles while transcription factors are represented as rectangles. Green arrows indicate that the nodes at each end of the arrow are regulated in the same direction (up or down) while the red arrows connect the nodes that are anticorrelated.

We hypothesized that decoding some of the important TFAs involved in B cells of MS patients might provide important clues to disease pathogenesis or uncover potential molecular targets for therapy. We started with an initial list of all genes changing at the 1.5-fold level. In this dual sample study, we assumed all pairs of genes moving in the same direction to be concordant and all pairs moving in opposite directions to be discordant. Combining this with the potential for regulatory relationships inferred from a TRANSFAC binding site dataset, we were able to extrapolate the potential transcription regulatory networks driving expression changes. We found that TCF2 and CDC5L bind very similar promoter motifs and seem to control overlapping sets of up and down regulated genes in this study. The transcripts we uncovered in this dataset are most notably, CCR1 and CXCR4, molecules which are associated with MS. The multiplication of corresponding cells in the TFBS and correlation matrices yields a regulation matrix where the sign of the interaction score reflects positive or negative regulation and the magnitude of the score reflects the amount of supporting evidence, as shown in the grid below (Figure 2). We examined these networks for various thresholds of fold-change and TRANSFAC evidence scores to obtain the picture of regulation of downstream molecules by tcf2 and cdcl5 shown in Figure 3.

## Results

**Figure 4:**
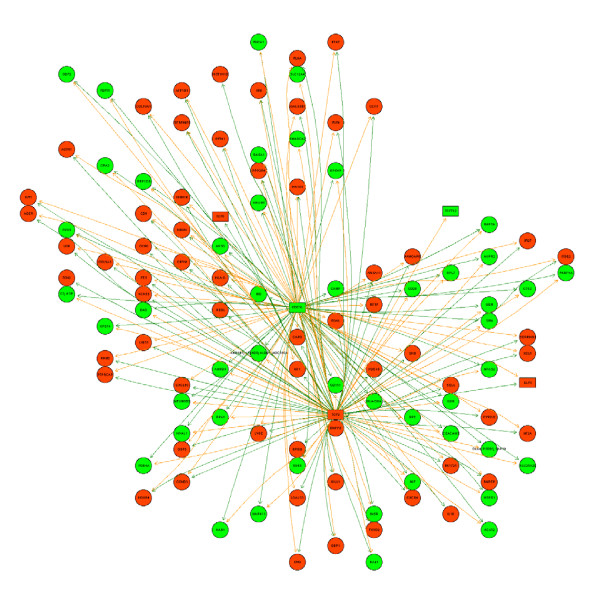
**Results from functional profiling of data using Onto-Express**. (A) The figure shows the significant (red) biological processes represented in a set of genes differentially expressed between two clinical groups. (B) The figure shows the significant (red) molecular function represented in a set of genes differentially expressed between two clinical groups.

As shown in Figure 1, the rows represent hierarchical clustering of all differentially expressed genes between the diseased (MS) and control (normal siblings) samples. The MS samples were 8923, 8922 A, 8830, 8839, 9013 and 9016, respectively. The unaffected siblings were 9017, 9018, 9023 and 8921A, respectively. Onto-Express analyses depicted in Figure 4 shows that transcription factor binding is a highly significant component in our microarray data. Results from Pathway-Express analysis are listed in Table 4, and these show that immune function and neurospecific pathways are also highly implicated in this data set.

**Table 4:** Results of pathway profiling using Pathway Express.

Rank	Database Name	Pathway Name	Impact Factor	#Genes in Pathway	#Input Genes in Pathway	#Pathway Genes on Chip	%Input Genes in Pathway	%Pathway Genes in Input	p-value
1	KEGG	Cell adhesion molecules (CAMs)	6.94	132	11	126	2.099	8.333	9.69E-04
2	KEGG	Axon guidance	6.94	130	11	126	2.099	8.462	9.69E-04
3	KEGG	Leukocyte transendothelial migration	5.288	117	9	113	1.718	7.692	0.005054
4	KEGG	Type I diabetes mellitus	5.232	44	5	40	0.954	11.364	0.005344
5	KEGG	Regulation of actin cytoskeleton	4.461	206	12	198	2.29	5.825	0.011556
6	KEGG	Natural killer cell mediated cytotoxicity	4.11	128	8	114	1.527	6.25	0.016401
7	KEGG	Focal adhesion	3.949	194	11	188	2.099	5.67	0.019276
8	KEGG	Tight junction	3.187	119	7	112	1.336	5.882	0.041314
9	KEGG	Antigen processing and presentation	3.112	86	5	68	0.954	5.814	0.044499
10	KEGG	Adherens junction	2.721	77	5	76	0.954	6.494	0.065823
11	KEGG	Colorectal cancer	2.676	77	5	77	0.954	6.494	0.068828
12	KEGG	Circadian rhythm	2.386	18	2	18	0.382	11.111	0.091979
13	KEGG	Maturity onset diabetes of the young	1.975	25	2	23	0.382	8	0.138816
14	KEGG	VEGF signaling pathway	1.972	72	4	70	0.763	5.556	0.139208
15	KEGG	Fc epsilon RI signaling pathway	1.83	75	4	74	0.763	5.333	0.160493
16	KEGG	mTOR signaling pathway	1.811	49	3	49	0.573	6.122	0.16353
17	KEGG	Huntington's disease	1.558	30	2	30	0.382	6.667	0.210455
18	KEGG	Wnt signaling pathway	1.474	147	6	144	1.145	4.082	0.229071
19	KEGG	MAPK signaling pathway	1.441	273	10	268	1.908	3.663	0.236637
20	KEGG	Toll-like receptor signaling pathway	1.416	91	4	88	0.763	4.396	0.242667
21	KEGG	B cell receptor signaling pathway	1.318	63	3	63	0.573	4.762	0.267565
22	KEGG	T cell receptor signaling pathway	1.294	93	4	93	0.763	4.301	0.274064
23	KEGG	Long-term potentiation	1.235	67	3	66	0.573	4.478	0.290928
24	KEGG	Insulin signaling pathway	1.143	135	5	131	0.954	3.704	0.318761
25	KEGG	Type II diabetes mellitus	1.117	44	2	41	0.382	4.545	0.327167
26	KEGG	Dentatorubropallidoluysian atrophy (DRPLA)	1.044	15	1	15	0.191	6.667	0.352194
27	KEGG	Epithelial cell signaling in Helicobacter pylori infection	0.997	46	2	45	0.382	4.348	0.368822
28	KEGG	Parkinson's disease	0.992	16	1	16	0.191	6.25	0.370684
29	KEGG	Cytokine-cytokine receptor interaction	0.963	256	8	241	1.527	3.125	0.381922
30	KEGG	Jak-STAT signaling pathway	0.936	153	5	144	0.954	3.268	0.392344
31	KEGG	Apoptosis	0.86	84	3	83	0.573	3.571	0.423249
32	KEGG	Complement and coagulation cascades	0.6	69	2	64	0.382	2.899	0.548544
33	KEGG	Olfactory transduction	0.523	31	1	31	0.191	3.226	0.592482
34	KEGG	SNARE interactions in vesicular transport	0.468	36	1	34	0.191	2.778	0.62642
35	KEGG	Phosphatidylinositol signaling system	0.421	79	2	78	0.382	2.532	0.656227
36	KEGG	Taste transduction	0.317	53	1	45	0.191	1.887	0.728441
37	KEGG	GnRH signaling pathway	0.277	97	2	95	0.382	2.062	0.758254
38	KEGG	Gap junction	0.27	99	2	96	0.382	2.02	0.763352
39	KEGG	Adipocytokine signaling pathway	0.145	69	1	69	0.191	1.449	0.864684
40	KEGG	Long-term depression	0.124	75	1	74	0.191	1.333	0.882976
41	KEGG	Neuroactive ligand-receptor interaction	0.102	302	5	279	0.954	1.656	0.902917
42	KEGG	ECM-receptor interaction	0.086	87	1	86	0.191	1.149	0.91743
43	KEGG	Cell cycle	0.042	112	1	110	0.191	0.893	0.958921
44	KEGG	Calcium signaling pathway	0.041	176	2	173	0.382	1.136	0.959904

We selected a subset of genes that were up and down regulated in the MS samples for further validation by RT-qPCR. Our results generally confirmed the results obtained with the Codelink arrays. TCF2, CXCL10, and FUT4 were all up regulated in the MS samples whereas CDC5L, TNFRSF19 and HLA-DR were down regulated (Table 5).

**Table 5:** Relative Expression of Selected Genes by RT-qPCR.

	Control		MS	
	RE	+/- SD	RE	SD

TCF2	1.00	0.68	7.95	4.25
CXCL10	1.00	0.47	5.93	3.85
FUT4	1.00	0.43	3.93	2.23
SERPIN B1	1.00	0.57	1.71	1.07
CDC5L	1.00	0.62	0.54	0.26
TNFRSF19	1.00	0.40	0.52	0.28
HLA-DRA	1.00	0.49	0.33	0.16

RE; relative expression.

+/-; calculated by determining relative expression for the value obtained by adding the ΔΔCt and ΔCt standard deviation (RE +/-), and then subtracting the RE +/- value from the RE.

For three other genes, we obtained disparate results for expression when the control and MS samples were pooled for analysis. CD83 was up regulated by RT-qPCR while down regulated by array analysis, SERPINB9 was down regulated by RT-qPCR while up regulated on the arrays, and CD9 was only minimally up regulated by RT-qPCR while clearly up regulated on the arrays (Table 6). For each of these genes, some samples were negative for expression by RT-qPCR (control sample 8921A for SERPINB9 and CD9, MS sample 8922A for CD83), and the influence of the negative values is not fully accounted for when pooling the samples prior to analysis by the ΔΔCt method. Thus, for these genes we further evaluated the RT-qPCR data by analyzing the control and MS samples individually and then averaging, rather than pooling the samples prior to the ΔΔCt analysis. After evaluating individual samples, we also found that some control samples were significant outliers for the CD83 and SERPINB9 genes. Control sample 8921A had a 26-fold increase in CD83 expression relative to the next highest control sample, and control sample 9018 had a 255-fold increase in SERPINB9 expression and a 156-fold increase in CD9 expression relative to the next highest samples for those genes (data not shown). To compensate for these outliers, we eliminated the highest expression samples from both the control and MS samples from the analysis and also included the negative samples. When analyzed in this way, CD83 expression in the MS samples was down regulated by 0.58-fold, SERPINB9 expression was up regulated by 2.47-fold, and CD9 expression was up regulated 3.5-fold relative to the Control samples (Table 6) and these results are in concordance with array data for these genes.

**Table 6:** Analysis of SERPINB9, CD83, and CD9 Expression by RT-qPCR.

	SERPINB9		CD83		CD9	
	Pool	Indiv	Pool	Indiv	Pool	Indiv
Control	1	1	1	1	1	1
MS	0.5	2.47	1.1	0.58	1.23	3.5

1. Pool; after RT-qPCR, Control and MS samples pooled prior to calculation of RE by ΔΔCt method.

2. Indiv; RE of samples first determined by ΔΔCtCt method, then Control and MS sample REs averaged (see text for details).

Although we included a Taqman assay for LYPLA3 in our validation studies, we were not able evaluate expression of this gene by RT-qPCR. None of the four Control samples were consistently positive for expression of this gene by RT-qPCR, and only one of the six MS samples was consistently positive. The probe for this gene on the Codelink array is located near the 3' end of the coding sequence whereas the Taqman assay detects sequences at the 5' end of the coding sequence, at the junction between exons 1 and 2 (6 total exons). Thus, full length cDNA would be required to detect LYPLA3 expression with the Taqman assay we employed. Our inability to consistently detect this gene suggests that we did not generate full length LYPLA3 cDNA during the RT step - either due to mRNA instability or inefficient reverse transcription.

### Additional data

A list of genes at 1.5 fold differential expression from microarray data can be found in Additional file 1.

## Discussion and Conclusion

The genes involved in our analyses encode proteins involved in apoptosis, cytokine pathways and inflammation. Although up or downregulation of gene transcription is not reflected in a 1:1 translation of protein expression, the gene product generally follows gene regulation dynamics [19]. A growing number of expression profiling studies provide experimental evidence indicating the presence of a transcriptionally distinct gene pattern in MS. Much of our current knowledge of MS stems from the analysis of a mouse model, experimental autoimmune encephalomyelitis (EAE) that is thought to be similar to MS. While EAE has striking clinical and histopathological similarities to MS, it has failed in predicting the efficacy of new therapeutics [20]. Our discussion is largely restricted to those genes that had > 2.5 fold up-regulation and the two transcription factors that had the most effect on other genes. Among the total list of differentially expressed genes, the proportion of downregulated genes was higher than that of upregulated ones (262 vs. 81), an observation that suggests an interplay involving complex inflammatory cascades in MS.

### Study limitations

The findings in our study are limited by a) sample size and b) the cellular pathways that Epstein-Barr virus (EBV) regulates remain poorly characterized and there are few data on how EBV may influence gene pathways in B cells derived from MS patients vs. controls. The ideal starting material would be to obtain native B cells from controls and MS patients; however, collection and processing bias are hard to eliminate in any microarray-based study.

### Upregulated genes

The gene for CXCL10 (interferon-γ-inducible protein-10) encodes for an interferon (gamma)-induced, secreted protein of 10 kDa, a chemokine of the CXC subfamily that is one of the ligands for the receptor CXCR3. The binding of this protein to CXCR3 causes pleiotropic effects, including stimulation of monocytes, natural killer and T-cell migration, and modulation of adhesion molecule expression (UCSC web browser). Its levels are increased in cerebrospinal fluid of MS patients with symptomatic attacks of demyelination, suggesting a role for this molecule in MS [21]. Following mouse hepatitis virus (MHV) infection in mice [22], CXCL10 (IFN inducible protein 10 kDa) protein was expressed during both acute and chronic stages of disease suggesting a role for this protein in disease exacerbation and chronicity. Previous studies have shown that during the acute phase of infection, T lymphocytes are recruited into the CNS by CXCL10. Our finding that CXCL10 upregulation (5.8-fold) in B cells from MS siblings is supportive of prior observations.

Serpin B1 is a leukocyte elastase inhibitor and regulates the activity of the neutrophil proteases elastase, cathepsin G and proteinase-3. In humans, serpins constitute 10% of the plasma proteins and are known as critical regulators of both thrombotic and fibrinolytic systems. Serpins participate in the regulation of the complement cascade, angiogenesis, apoptosis and innate immunity. Most of the human clade B serpins inhibit serine and/or papain-like cysteine proteinases and protect cells from exogenous and endogenous proteinase-mediated injury. As some serpins also guard cells against the deleterious effects of promiscuous proteolytic activity [23], it is possible that the cytoprotective function is a common feature of intracellular serpin clades and we hypothesize that up-regulation of serpin B1 gene may be protective in MS. While our findings need to be validated, serpin B1 gene may represent a novel therapeutic target to ameliorate MS, considering the importance of these molecules in regulating proteolytic cascades.

We found that FUT4 gene was upregulated by 2.5 fold. It is a member of the interleukin 1 cytokine family and the protein encoded by the gene is produced by activated macrophages as a pro-protein and processed to its active form by caspase 1 (CASP1/ICE). This cytokine is an important mediator of the inflammatory response, cell proliferation, differentiation, and apoptosis. The induction of cyclooxygenase-2 (PTGS2/COX2) by this cytokine in the central nervous system (CNS) is found to contribute to inflammatory pain hypersensitivity. FUT4 mRNA is increased in Jurkat cells undergoing apoptosis.

LYPLA3 (lysosomal phospholipase A2): Lysophospholipases are enzymes that act on biological membranes to regulate the multifunctional lysophospholipids. The protein encoded by this gene hydrolyzes lysophosphatidylcholine to glycerophosphorylcholine and a free fatty acid. LYPLA3 is present in the plasma and thought to be associated with high-density lipoprotein. Cellular phospholipases are key participants in cellular transduction [24] and are thought to be involved in the pathogenesis of local and systemic inflammatory disorders [25]. Interestingly, cytosolic phospholipase A2 levels were found to be low, possibly reflecting proteolysis or inactivation of enzyme activity in brain MS lesions [26].

The gene for XCL1 was upregulated; it encodes for chemokines, a group of small (8-14 kD) molecules that regulate cell trafficking of leukocytes through interactions with a subset of 7-transmembrane, G protein-coupled receptors. Chemokines also play fundamental roles in the development, homeostasis, and function of the immune system, and they have effects on cells of the central nervous system as well as on endothelial cells involved in angiogenesis or angiostasis. The protein product of XCL1 has chemotactic activity for neutrophils may play a role in inflammation and exerts its effects on endothelial cells in an autocrine fashion. We also found PSTPIP1 (proline-serine-threonine phosphatase interacting protein1) upregulated; its role in MS is unclear.

### Downregulated genes

An important finding in our study is an 11.7-fold downregulation of eukaryotic translation initiation factor 1A, Y-linked (EIF1AY) gene. It is thought that EIF1AY encodes for minor histocompatibility antigen (mHA) and B-cell mediated antibody response to Y-chromosome encoded histocompatibility antigens (H-Y antigens) is associated with maintenance of disease remission in graft-vs-host disease [27]. The B cells may provide a new target for immune intervention in chronic graft vs. host disease and whether such a correlation can be translated to chronic MS requires further study.

We found that the tumor necrosis factor receptor (TNFR) superfamily 19 gene (TNFRSF 19) was downregulated by 5.3-fold. In a recent study by Achiron et al., TNF superfamily members 14, 1B, 4, and 6 were downregulated. The TNFR superfamily of cell surface receptors share approximately 80 amino acids within their cytoplasmic region called the death domain (DD), critical for recruiting the death machinery [28]. Individual TNF receptors are expressed in different cell types and have a range of affinities for various intracellular molecules that provide signaling and biological specificities. However, much is unknown about how individual signal transduction pathways are regulated upon activation by any particular TNF receptor, under physiological conditions [29] and while TNFR superfamily 19 genes may mediate important functions in immunity, inflammation, differentiation, cell proliferation control, and apoptosis, their specific effects in MS are unknown.

The gene for CD83 was downregulated in MS siblings. CD83 molecules are expressed at a high level on immune-competent, activated and mature dendritic cells. In MS, CD83-positive dendritic cells are present in occasional perivascular cuffs in lesions, suggesting that mature dendritic cells migrate to the CNS in response to chemokine signals [30].

The downregulation of insulin receptor tyrosine kinase substrate (BAIAP2L1) gene in B cells of MS siblings is significant. The insulin-like growth factor I receptor (IGF-IR), is a member of the receptor tyrosine kinase family of growth factor receptors. In rheumatoid arthritis (RA) it has been shown that IgG antibodies from the sera of patients can stimulate synovial fibroblasts through interaction with insulin-like growth factor receptor 1 (IGF-R1), provoking trafficking of T cells [31]. The data demonstrate, for the first time, a bridging link between B-cell activity and T-cell trafficking. In addition, they are of potential importance for the development of innovative therapeutic strategies, in which interrupting the IGF-1/IGF-1R axis could result in sustained disease modification by affecting both the growth-factor triggered activation of fibroblasts and the accumulation of T lymphocytes. A similar link in MS has not been demonstrated but is plausible. Other genes downregulated include ADAMTS16, BID, MIF and DAPK2, involved in the apoptosis pathway.

### TCF2, diabetes and MS

Our transcription and microarray analysis showed that TCF2 was upregulated. TCF2 encodes transcription factor 2, a liver-specific factor of the homeobox-containing basic helix-turn-helix family. The TCF2 protein forms heterodimers with another liver-specific member of this transcription factor family, TCF1; depending on the TCF2 isoform, activation or inhibition of transcription of target genes occurs. We found that TCF2 upregulates the CC chemokine receptor 1 (CCR1) a molecule expressed on macrophages and T cells; in MS plaques, numerous CCR1-positive infiltrating macrophages and microglial cells, associated with CCL3, were found [2] and CCR1 has been associated with newly infiltrating monocytes in MS lesions [32]. CCR1-deficient mice show an attenuated EAE course [33]. Additionally, CXCR1, a pro-inflammatory molecule is upregulated by TCF2. Furthermore, TCF2 downregulated CD28, a co-stimulatory molecule involved in T-cell stimulation and IL6R, a pro-inflammatory molecule. Taken together, it is plausible that TCF2 plays a central role in the pathogenesis of MS.

Interestingly, a mutation in TCF2 has been linked to the etiology of MODY5 (Maturity-Onset of Diabetes, Type 5) and in a recent Danish nationwide cohort study, intra-individual and, to a lesser degree, intra-familial co-occurrence was evident in MS and type 1 diabetes. The underlying mechanisms may involve both genetic and environmental factors [34]. Individuals with type 1 diabetes are more than three times more likely to develop MS than controls; in addition, the two diseases appear to be linked, albeit to a weak extent, within families. The exact mechanism of how mutations in these transcription factors cause diabetes remains unknown. To reiterate, a link between TCF2 mutation and MODY5 has been established, while type 1 diabetics have an increased risk of MS development. Our study points to TCF2 having a prominent role in regulation of other transcription factors. In summary, the role of TCF2 in MS needs to be further explored since MODY5 and TCF2 are linked and type 1 diabetes confers increased risk of MS development.

Another transcription factor that we found in our transcription analysis, CDC5L, was downregulated. It is a well-characterized pre-mRNA splicing factor and involved in cell cycle kinetics in yeast, S. pombe; its role in MS, if any, is not characterized.

## Competing interests

The author(s) declare that they have no competing interests.

## Authors' contributions

JRA (Jagannadha R Avasarala) designed, conceptualized the study format and wrote the manuscript; SVC (Sridar V Chittur) did the microarray studies, analyzed the data and authored the materials and methods section; JAT (John A Tine) did quantitative RT-PCR while ADG (Ajish D George) performed the transcription factor network analysis. All authors have read and approved the final version of the manuscript.

## Pre-publication history

The pre-publication history for this paper can be accessed here:



## Supplementary Material

Additional file 1 List of genes at 1.5 fold differential expression from microarray data. The data provided represents that complete list of statistically significant genes from the microarray analysis, which show differential expression at a 1.5 fold cut-off. Click here for file
